# A Polydopamine-Functionalized Carbon Microfibrous Scaffold Accelerates the Development of Neural Stem Cells

**DOI:** 10.3389/fbioe.2020.00616

**Published:** 2020-06-23

**Authors:** Yanru Yang, Yuhua Zhang, Renjie Chai, Zhongze Gu

**Affiliations:** ^1^State Key Laboratory of Bioelectronics, Southeast University, Nanjing, China; ^2^Key Laboratory for Developmental Genes and Human Disease, Ministry of Education, Institute of Life Sciences, Southeast University, Nanjing, China; ^3^Co-innovation Center of Neuroregeneration, Nantong University, Nantong, China; ^4^Institute for Stem Cell and Regeneration, Chinese Academy of Science, Beijing, China; ^5^Jiangsu Province High-Tech Key Laboratory for Bio-Medical Research, Southeast University, Nanjing, China

**Keywords:** three-dimensional scaffolds, biomaterials, polydopamine, carbon microfiber, neural stem cell

## Abstract

Neuroregenerative medicine has witnessed impressive technological breakthroughs in recent years, but the currently available scaffold materials still have limitations regarding the development of effective treatment strategies for neurological diseases. Electrically conductive micropatterned materials have gained popularity in recent years due to their significant effects on neural stem cell fate. Polydopamine (PDA)—modified materials can also enhance the differentiation of neurons. In this work, we show that PDA-modified carbon microfiber skeleton composites have the appropriate conductivity, three-dimensional structure, and microenvironment regulation that are crucial for the growth of neural stem cells. The design we present is low-cost and easy to make and shows great promise for studying the growth and development of mouse neural stem cells. Our results show that the PDA-mediated formation of electrically conductive and viscous nanofiber webs promoted the adhesion, organization, and intercellular coupling of neural stem cells relative to the control group. PDA induced massive proliferation of neural stem cells and promoted the expression of Ki-67. Together, our results suggest that the composite material can be used as a multifunctional neural scaffold for clinical treatment and *in vitro* research by improving the structure, conductivity, and mechanical integrity of the regenerated tissues.

## Introduction

Neurodegenerative diseases lead to severe sensory and motor damage in the human body and are among the leading causes of social disability and death globally, and the lack of transplantable nerve tissue is the prime hindrance to clinical nerve repair. Due to their multipotency and self-renewal, neural stem cell (NSC) transplantation therapy is one of the most promising strategies for treating traumatic injuries and neurodegenerative disorders. *In vitro* NSC expansion (to increase NSC resources) through nerve tissue engineering (NTE) methods might provide the most effective way to solve this clinical shortage. The ideal tissue engineered nerves are composed of scaffold materials, a microenvironment that induces or promotes cell growth, and NSCs. Biomaterials and their biocompatible modifications, for example, oriented substrates like electrospun microfibers, conductive materials such as graphene, and ordered materials like photonic crystals are gaining widespread use in the field of NTE applications because the scaffolds have shown the unique characteristics of topological cues and bioelectric properties for mimicking the extracellular matrix (ECM) or for performing other functions (Martino and Pluchino, 2006; He et al., 2012; Limongi et al., 2018).

In recent years, electrospinning technology has generated considerable interest in fabricating tissue engineering biomaterials, mainly due to the morphological and structural properties of such materials, which almost perfectly mimic the fibrillary 3D architecture of the natural ECM (Carlberg et al., 2009; Zhu et al., 2018). Furthermore, an increasing body of evidence suggests that NSC fate—including cell adhesion, differentiation, migration, and proliferation—is greatly affected by the pattern of biochemical, physical, and topographical properties that constitute the essential elements of their niches *in vivo* (Kam et al., 2008; Heo et al., 2011; Yang et al., 2012; Ma et al., 2016). The electrospun scaffolds with aligned fibers display the versatility of ECM mimicry, which required in NTE, and several studies have been performed using this feature of electrospun fibers to induce the differentiation of NSCs. Gregory et al. fabricated electrospun polyethersulfone fiber mesh to study the influence of different fiber diameters of electrospun substrates on rat hippocampus-derived adult NSC differentiation and proliferation (Christopherson et al., 2009). Qi et al. produced a versatile substrate for insulin-like growth factor 1 delivery in a graphene oxide-incorporated poly (lactic-co-glycolic acid) electrospun nanofibrous mat and found improved NSC survival, diffusion, and differentiation (Qi et al., 2019). The electrospun micro- or nanofibers that the cells are interfacing with influence NSC growth, and the physical properties of the spun materials (such as electrical conductivity, mechanical strength, etc.) guide the development of NSCs. Neurons are electroactive cells, and cell dysfunction is also closely related to electrical activity. Electrical stimulation can induce depolarization of excitatory cell membranes and induce functional neuronal responses, and thus conductive materials could be a promising option for applications in NTE. To date, conductive biomaterials have been widely used for tissue engineering, such as they can apply in skin wound healing (He et al., 2000), 3D myoblast guidance (Wang et al., 2015), peripheral nerve tissue engineering (Wu et al., 2016) and engineered 3D cardiac anisotropy (Wu et al., 2017) and so on. Neuronal cells are quickly excited and transmit electrical signals in one direction, and the use of conductive electrospun nerve scaffolds to promote autologous nerve repair has become a trend in recent years. The electrical stimulation on theses scaffolds might also be an important factor for facilitating neurite extension and differentiation (Koppes et al., 2016; Granato et al., 2018; Uz and Mallapragada, 2019). Cellulose has excellent conductivity after carbonization, so it is a great original material for conductive electrospinning technology.

Polydopamine (PDA) is a highly viscous and highly adsorbent polymer membrane alkaline formed by the self-polymerization of the neurotransmitter dopamine. PDA has the characteristics of a polyelectrolyte in a primary environment due to the deprotonation of the catechol group. Thus, in the presence of alkaline *p*H, an electrostatic interaction is generated between the PDA and cationic polyelectrolytes. Alternating deposition on the surface of the material allows PDA to self-assemble the biomaterials layer by layer (Lee et al., 2007; Wei et al., 2010; Faure et al., 2013). PDA can form a tightly adhering composite layer on the surface of almost any solid material, and this one-step modification is efficient, versatile, cost-effective, and environmentally friendly. Thus, PDA-mediated surface coating methods can be used to modify a wide variety of biomaterials for a diverse array of functional applications in stem cell-based tissue engineering. As an example, Wu et al. demonstrated that a PDA-based coiled polycaprolactone scaffold could promote mesenchymal stem cell differentiation (Shin et al., 2011). Another group studied the combined effects of PDA functionalization and collagen on micro-grated polydimethylsiloxane and showed that the material improved the culture of human mesenchymal stem cells (Sharma et al., 2019). These studies have shown that PDA-modified biomaterials can stimulate neurons and guide axonal extension even in the absence of some functions such as conductivity, topography, and microenvironment control. However, as Yang et al. (2018) proposed, the existing studies have not considered adding such factors together to regulate NSC growth behavior and thus identify more efficient and economical methods for use in NTE. Therefore, the combination of a PDA functionalized scaffold and carbonized electrospun fiber with unique electrochemical properties might be a new strategy for the design of biomaterials and microenvironments for NTE.

The goal of this study was to develop an electrically conductive and adherent 3D scaffold and to examine the effect on NSC development by combining the effects of PDA functionalization and electrical stimulation with 3D structures. Our results indicate that compared to the untreated carbon fiber (CF) group, the PDA-mediated collection of electrically conductive and viscous microfiber webs improved the adhesion, organization, and intercellular coupling of NCSs. The presence of PDA resulted in a significantly increased proliferation of NSCs and increased expression of Ki-67. Representative 3D reconstructions of confocal microscope images demonstrated that NSCs quickly grew into the interior of the PDA-CF but only accumulated across the surface of the CF scaffold. The expression of vinculin by NSCs was also significantly enhanced in NSCs grown on PDA-CF compared to those grown on CF. These results indicate that PDA promotes the adherence and proliferation of NSCs on the conductive 3D CF skeleton and thus that PDA-CF might be used to prepare multifunctional neuron scaffolds for both treatments and for *in vitro* research.

## Materials and Methods

### Fibrous Scaffold Fabrication

To prepare microfibers, surgical cotton batting (DP of 1140) was ultrasonically washed with absolute ethanol for at least 2 h and dried under vacuum at 50°C and then ground into a powder. The electrospinning solution (1.1 wt%) was prepared by dissolving the fine cellulose particles in lithium chloride/N, N-dimethylacetamide (Kim et al., 2005) for 12 h at room temperature. The cellulose solution was loaded into a 30 ml syringe and pushed through a 0.86 mm inner diameter stainless-steel needle with a set flow rate of 0.2 ml/min and a 20-kV high-voltage source. The distance between the needle and the collector was set at 20 cm. The electrospun fibrous scaffolds were finally peeled off from the screen (the aluminum foil), run through a solid-phase extraction column (Dong qi Bio-technology Co., China), and dehydrated in a CO_2_ critical point dryer (HCP-2, Hitachi Ltd) before subsequent carbonization.

### Characterization of the CF Scaffold

The physicochemical properties of the surfaces of the tested materials were determined by Raman spectrometry (RAMAN, inVia, Renishaw Inc.) with a 532 nm laser. To conduct the wettability test, the samples were placed on a microscope slide and pressed slightly to smooth the surface and the contact angle was measured (JC2000D, Powereach, China). The morphological features of the materials were determined by sputter coating with a gold layer using a Hitachi E-1010 ion sputter (Hitachi, Ltd., Japan) and then imaging by scanning electron microscopy (SEM, Zeiss Ultra Plus, Germany). The elemental composition of the material was determined in real-time using an X-Max Energy Dispersive Spectrometer (EDS). The particular PDA-CF and CF groups of the sample were identified by Vertex 70-type Fourier-transform infrared spectroscopy (FT-IR). A 5–10 mg sample was mixed with potassium bromide and pressed into thin slices, and the test was performed in transmissive mode with 32 scans.

### Isolation and Culture of NSCs

The NSCs were isolated from the hippocampus zones of embryonic day 18.5 wild-type mouse brains. The hippocampus was removed and collected in a 1.5 ml centrifuge tube and then dissociated by Accutase (Gibco, USA) for 25 min in a 37°C incubator. The tissue was triturated mechanically by using pipet tips. NSCs were suspended in DMEM-F12 medium (Gibco, USA) containing 2% B-27, 20 ng/ml EGF, and 20 ng/ml FGF (R&D Systems, USA) and cultured in a humidified atmosphere with 5% CO_2_ at 37°C. The passage of NSCs was performed every 7 days during culturing. Before seeding, the CF and PDA-CF scaffolds were coated with laminin (10 μg/ml, 37°C overnight). After purifying three generations, the NSCs for the proliferation studies were seeded at a concentration of 5 × 105 cells/ml in proliferation medium consisting of DMEM-F12 medium with 2% B-27 supplemented with 20 ng/ml EGF and 20 ng/ml FGF. For the differentiation studies, NSCs were seeded at a similar concentration in DMEM-F12 medium containing 2% B-27 supplemented with fetal bovine serum (GIBCO, USA) and 1 μM retinoic acid (Sigma). The care and use of animals in these experiments followed the guidelines and protocols approved by the Care and Use of Animals Committee of Southeast University. All efforts were made to minimize the number of animals used and their suffering.

### SEM Observation of NSCs Cultured on PDA-CF

After culturing for 5 days, NSC cultures were washed with PBS two times and then fixed with 2.5% glutaraldehyde overnight at 4°C. The samples were dehydrated by an ethanol gradient series (30, 50, 70, 80, and 90%), lyophilized, sputter-coated with gold for 2 min, and then analyzed by SEM (Zeiss Ultra Plus, Germany).

### Cell Viability/Proliferation Assay

Cell viability and proliferation were assessed by CCK-8 assay. After culturing in proliferation media for 3 days, NSCs were incubated with CCK-8 reagent (diluted 20-fold in culture medium) for 2 h before the media were collected. The tetrazolium salt in CCK-8 is reduced by dehydrogenase in the cytoplasm to produce a yellow color. The intensity of the yellow color—which corresponds to the number of viable cells—was analyzed by a plate reader at 450 nm.

At the same time, after 5 days of culture cell viability was tested using the LIVE/DEAD viability/cytotoxicity kit for mammalian cells (Invitrogen, USA) according to the manufacturer's instructions. The percentage of living cells was calculated by determining the percentage of calcein-AM–positive cells out of the total cell number.

### Immunofluorescence Staining of Cultured NSCs

NSCs were washed with PBS and fixed in 4% paraformaldehyde (PFA) for 30 min at room temperature, followed by blocking with PBS (pH 7.4) containing 5% donkey serum, 0.1% Triton X-100, 0.02% sodium azide (NaN_3_), and 1% bovine serum albumin for 1 h at room temperature. This was followed by overnight incubation with primary antibodies at 4°C. Immunostaining was resumed by washing with PBS and incubating with secondary antibody (Invitrogen) for 1 h at room temperature followed by washing and then mounting in antifade fluorescence mounting medium (DAKO). The antibodies used in this study were anti-Ki-67, anti-TuJ1, and anti-nestin (Abcam, USA). Cell proliferation was measured with the Click-it EdU imaging kit (Invitrogen). Three independent experiments were repeated for each assay.

### RNA Extraction and RT-PCR

The RNA isolation, reverse transcription, and PCR analysis were performed following standard protocols. Briefly, total cellular RNA was isolated using TRIzol reagent (Life Technologies), 1 μg of RNA was reverse transcribed using TaqMan Reverse Transcription Reagents (Applied Biosystems), and the mRNA levels of the indicated genes were measured in triplicate using the SYBR Green master mixture (Applied Biosystems) and a Chromo-4 real-time RT-PCR instrument (MJ Research). The mRNA levels were normalized to GAPDH (internal control), and gene expression was presented as fold changes (Ct method).

### Statistical Analysis

All experiments were repeated three times in triplicate for each assessment, and all data are expressed as the mean ± standard deviation. Data were analyzed by one-way by Tukey's tests, and a *p* of < 0.05 was considered statistically significant.

## Results and Discussion

### Fabrication and Characterization of the PDA-CF Scaffold

**Figure 1:**
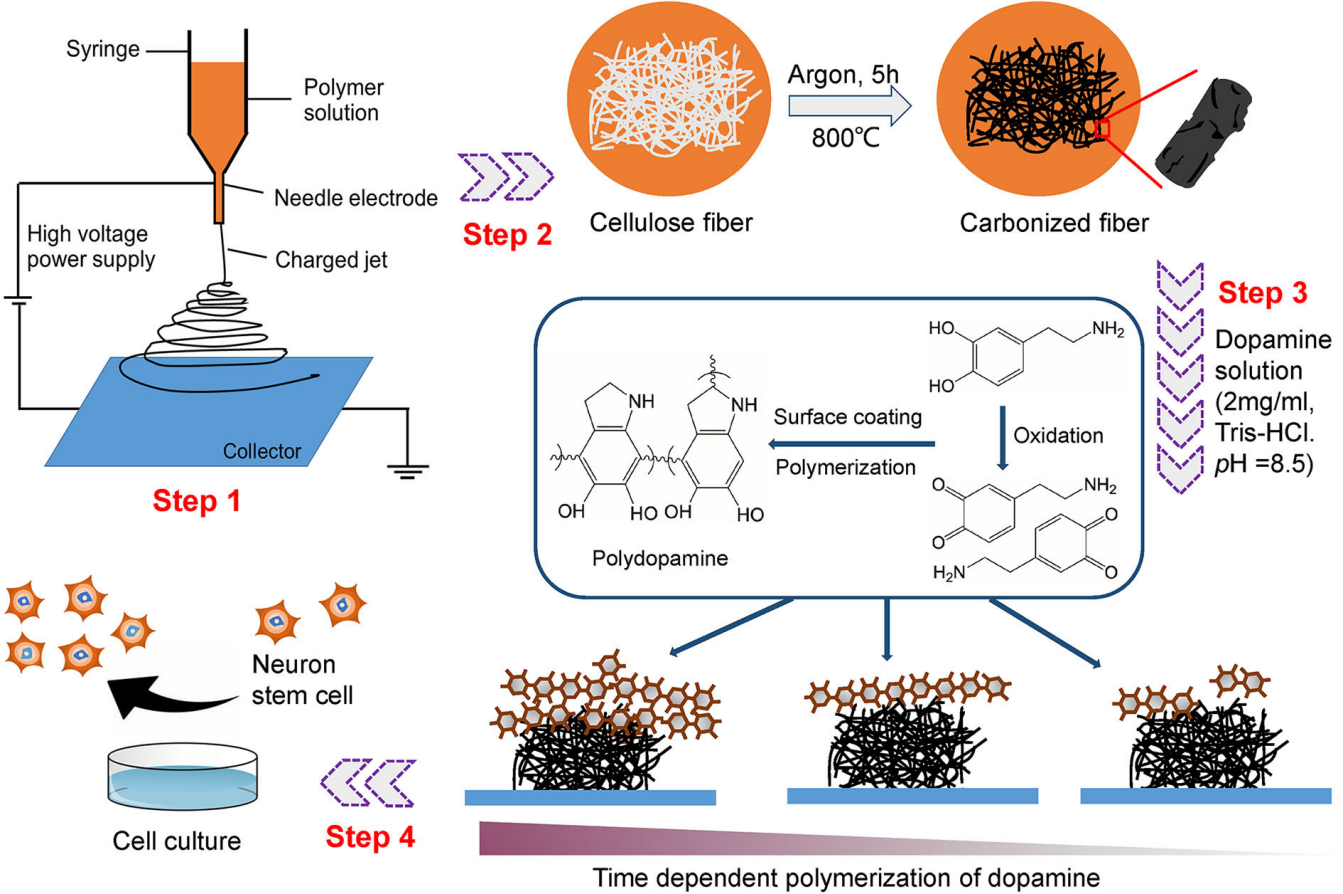
Schematic diagram illustrating the preparation of the 3D PDA-CF used for NSC culture.

**Figure 2:**
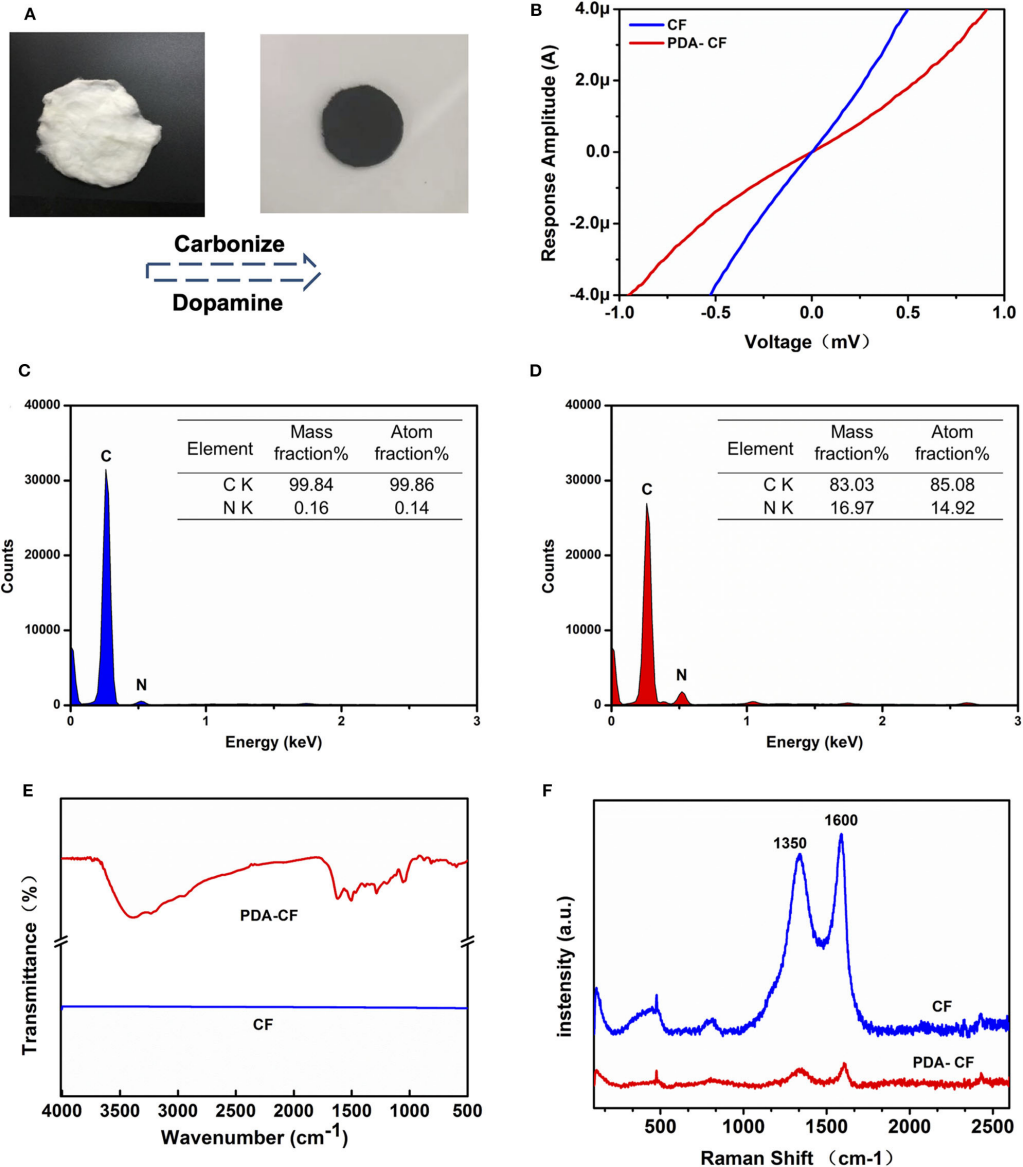
Characterization of the PDA-CF scaffold. **(A)** Cellulose fiber (left) and the fixed PDA-CF (right) after carbonization and dopamine polymerization treatment. **(B)** I-V curve indicated that the PDA-CF has advanced electrophysiological function. **(C,D)** The EDS spectral analysis. **(E)** FT-IR spectra of the samples. **(F)** Raman spectra of the samples using an incident laser wavelength of 632.8 nm.

Figure 1 shows the overall procedure for the fabrication of the PDA-CF scaffold and its application in NSC culture. First, the cellulose microfibers were fabricated from natural cellulose by electrospinning, and the cellulose fibers formed carbon aerogels after high-temperature carbonization (heat treatment in the tube furnace at 800°C for 5 h). The synthesis of PDA is depicted in step 3. Dopamine (2.0 mg/ml) was used as the precursor, and CFs doped with PDA self-assembled under alkaline conditions. The carbonized fibers show excellent flexibility which can be bent into different angles and folded as Li et al. (2016) presented in their study, the material was finally fixed on a circular cover glass with a diameter of 1 cm (Figure 2A).

**Figure 3:**
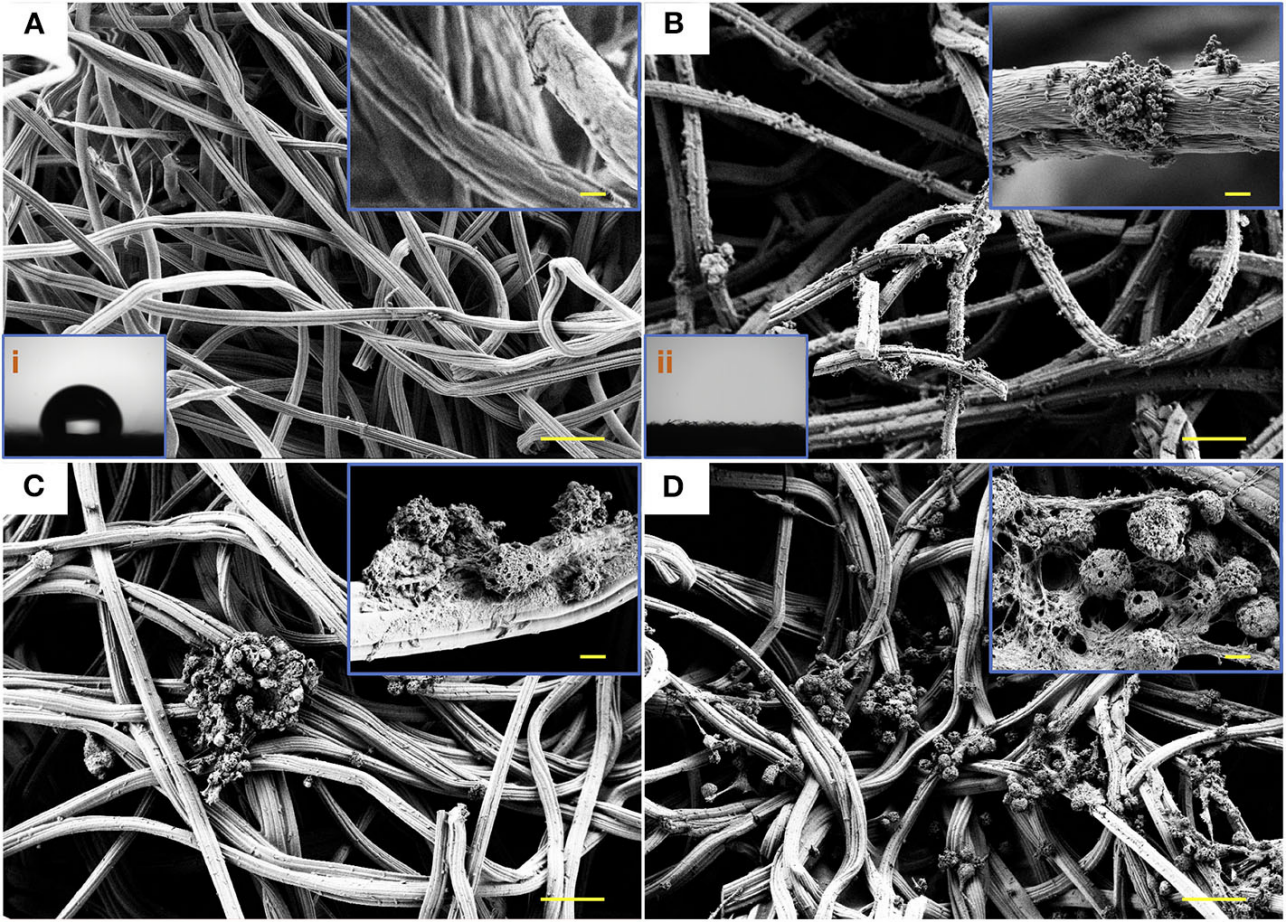
SEM micrographs and water contact angle. SEM images illustrating the networked surfaces of CF **(A)** and PDA-CF **(B)** and the water contact angle (i and ii) of the two materials. The growth of NSCs cultured on CF **(C)** and PDA-CF **(D)**. Scale bars in the high-magnification images are 20 μm, and in the low-magnification images are 0.5 μm.

The morphological features of the tested materials before and after dopamine treatment were characterized by SEM. The CFs (Figure 3A) retained almost all the topological cues from electrospinning before thermal annealing. In the image of PDA-CF (Figure 3B), it can be seen that the CF surface was very rough and that the fibers were covered with clusters of particles that we speculated were the PDA layer. Further evidence for this came from the EDS analysis and images (Figures 2C,D). The carbon content of the untreated CF was 99.84%, and the nitrogen content was only 0.16%. After dopamine treatment, the nitrogen content increased to 16.97%, and because the additional nitrogen mainly derived from the PDA layer, this indicated that PDA had effectively been deposited onto the CF.

FT-IR (Figure 2E) also illustrated this point. The analysis of the material by infrared spectroscopy showed a straight line for CF with no absorption vibration peaks. This result is consistent with the characteristics of the material itself because the cotton cellulose material is a pure carbon sample after high-temperature carbonization. The infrared absorption of PDA-CF varied between 1200 and 1600 cm^−1^. According to the literature, 1512 cm^−1^ is the absorption peak of N–H, and 1284 cm^−1^ is the tensile vibration peak of a typical polymer C=O, both of which are characteristic absorption peaks in the PDA structure (Kim et al., 2005; Wang et al., 2008; Tang et al., 2015; Li et al., 2016). Taken together the results of SEM, EDS, and FI-IR analysis confirmed that PDA successfully coated the surface of the CFs.

The Raman analysis (Figure 2F) showed two peaks for graphite, one at 1,596 cm^−1^ corresponding to the G band and one at 1,350 cm^−1^ corresponding to the D band (Li et al., 2016). The conductivity test (Figure 2B) showed that the PDA-CF was electrophysiologically functional. Furthermore, we calculated the resistance from the I-V curve of the CF and PDA-CF scaffolds and found that the PDA-CF had a greater resistance (around 1,200 Ω in an area of 500 mm × 500 mm) compared to the CF (540 Ω). The main reason for this difference might be that the adhesion of the PDA reduces the conductivity of the material. As carbonized cellulose microfibers are in highly dense and hydrophobic before PDA modification, the solution is not entirely covering the surface, leading to the PDA layer coat on CF as particle clusters, which is the best method to protects the conductive properties of the materials. We further analyzed the water contact angle [Figures 3A(i),B(ii)] on the CF and PDA-CF substrates. Due to the hydrophobic nature of the CF material itself, the water does not wet it and instead forms beads on the surface of the sample. In contrast, water droplets on the PDA-CF surface quickly penetrate the sample layer, and no beads of water are visible on the surface of the sample. Thus, the PDA modification transforms the relatively hydrophobic CF material into a highly hydrophilic material, which is beneficial for the adhesion and growth of cells.

### The Biocompatibility of PDA-CF

We isolated and amplified NSCs from the hippocampus of fetal mice and seeded them on the PDA-CF and CF scaffolds. In most of the SEM observations, as illustrated in Figures 3C,D, the PDA-CF scaffold seemed to enhance NSC proliferation. The calcein acetoxymethyl ester (calcein-AM) and ethidium homodimer-1 (EthD-1) staining assays were used to evaluate the cytotoxicity of PDA-CF. As shown in Supplementary Figure 1A, almost 83% of the cells cultured on PDA-CF for 5 days were alive, and there were significant differences between PDA-CF and CF in terms of cell viability (Supplementary Figures 1B,C), thus suggesting that PDA-CF has exceptional biocompatibility.

**Figure 4:**
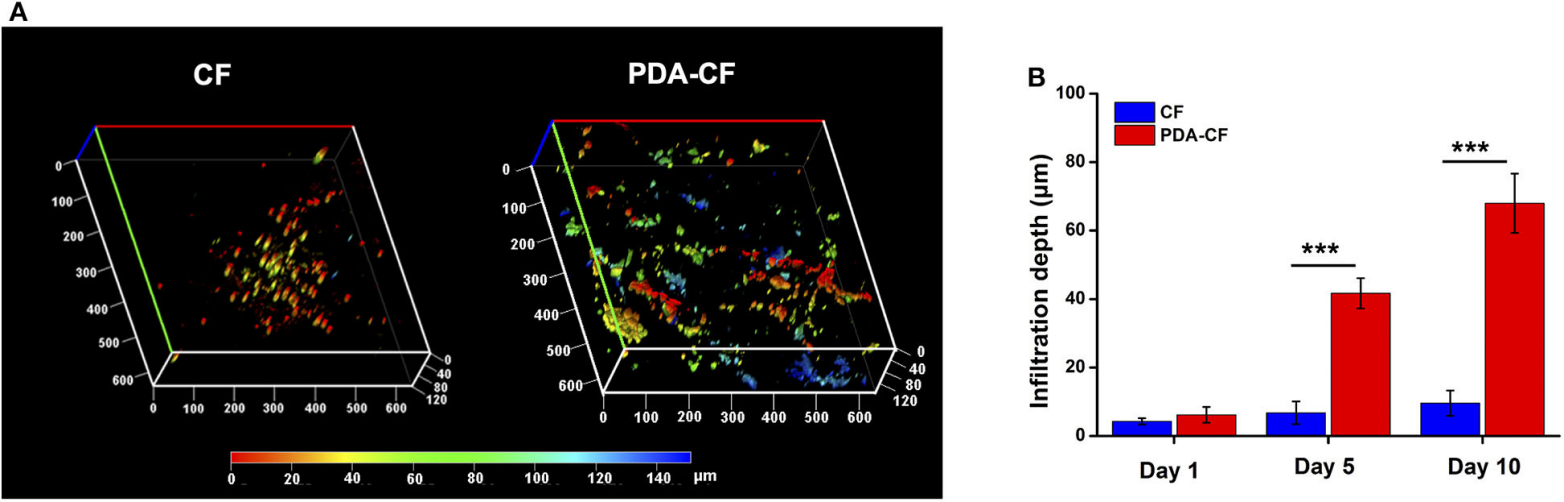
Representative 3D reconstructions of confocal laser scanning microscopy mapping **(A)** after 5 days of culture. The depth of cell growth on the materials can be seen from the z-axis. **(B)** Mean infiltration depths on the CF and PDA-CF scaffolds (*p < 0.05; **p < 0.005; ***p < 0.0005).

The cells were stained with DAPI after culturing on the scaffolds for 1 day, 5 days, and 10 days, and representative 3D reconstructions were made of the confocal microscope images with different colors showing cells at different depths. From the z-axis, we found that NSCs grew quickly into the interior of the PDA-CF after 5 days of cell culturing (Figure 4A), while NSCs primarily expanded along the surface of the CF scaffold. We also measured the cell infiltration depth on the two materials (Figure 4B), and the results were consistent with what we observed directly from the images. The greater infiltration of NSCs grown on PDA-CF suggests that PDA-CF might promote cell migration and cell viability.

### NSC Proliferation on PDA-CF

**Figure 5:**
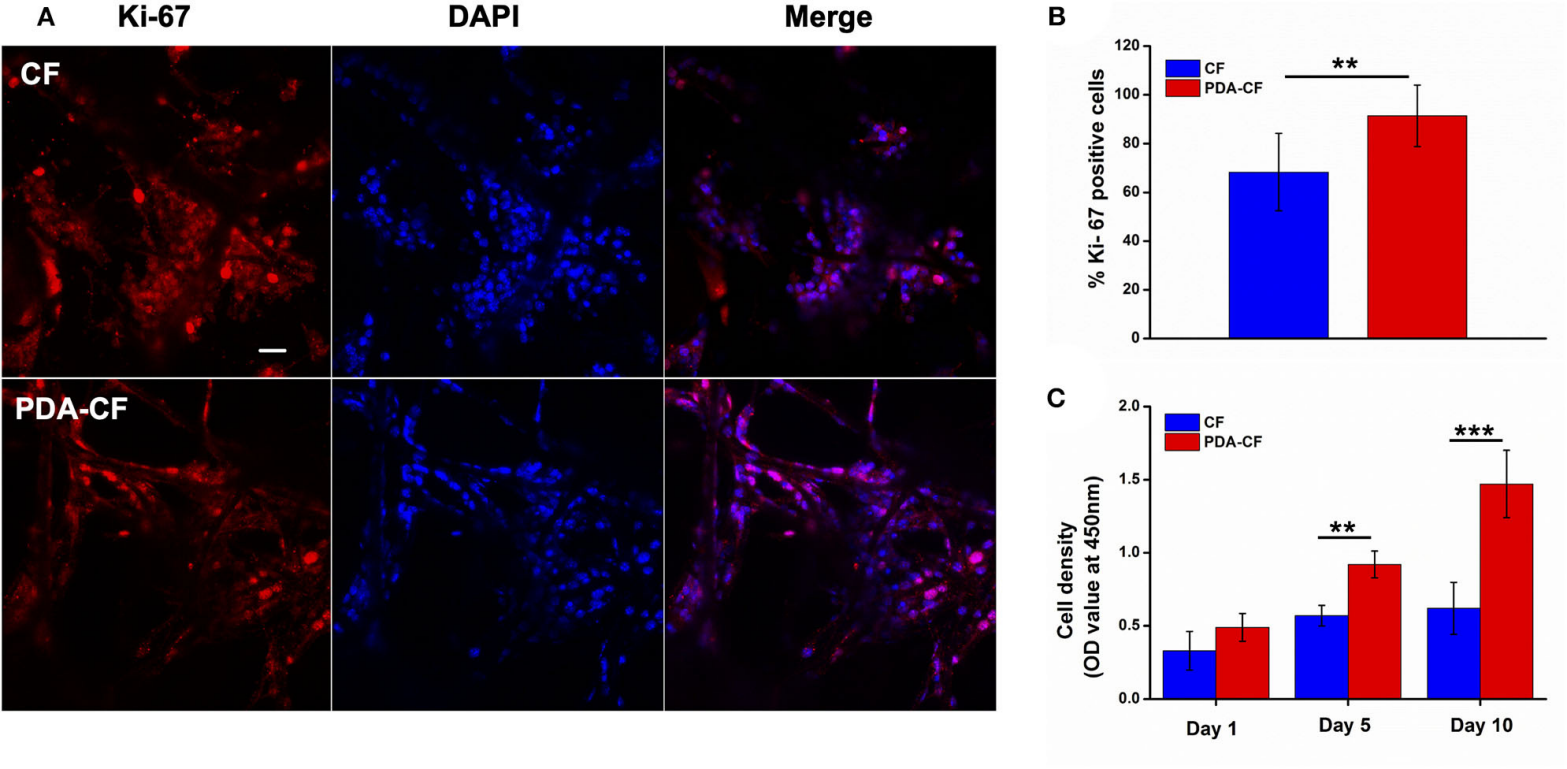
PDA-CF increased the proliferation of NSCs compared to the CF control. **(A)** Laser scanning confocal microscopy in NSCs showing immunostaining of Ki-67 and DAPI staining. **(B)** The proportions of Ki-67–positive cells after 5 days of culture. **(C)** CCK-8 analysis of relative cell viability. Scale bar is 50 μm (**p < 0.005; ***p < 0.0005).

The effect of PDA-CF on NSC proliferation was assessed by Ki-67, nestin, and 5-ethynyl-2′-deoxyuridine (EdU) immunofluorescence staining. The Ki-67 protein, which is required for cell division, is an active mitotic marker of proliferating cells during neurogenesis. Nestin is the intermediate filament protein for labeling and identifying NSCs. EdU is a thymidine analog that is incorporated into replicating chromosomal DNA during the S phase of the cell cycle and is widely employed to measure cell proliferation in the nervous system (Scholzen and Gerdes, 2000; Chehrehasa et al., 2009; Park et al., 2010). The expression of Ki-67 protein after 5 days of culture is presented in Figure 5A, and quantification (Figure 5B) showed that NSCs cultured on PDA- CF had a greater number of Ki-67–positive cells. The CCK-8 results (Figure 5C) suggested that PDA-CF could promote cell proliferation better than CF.

**Figure 6:**
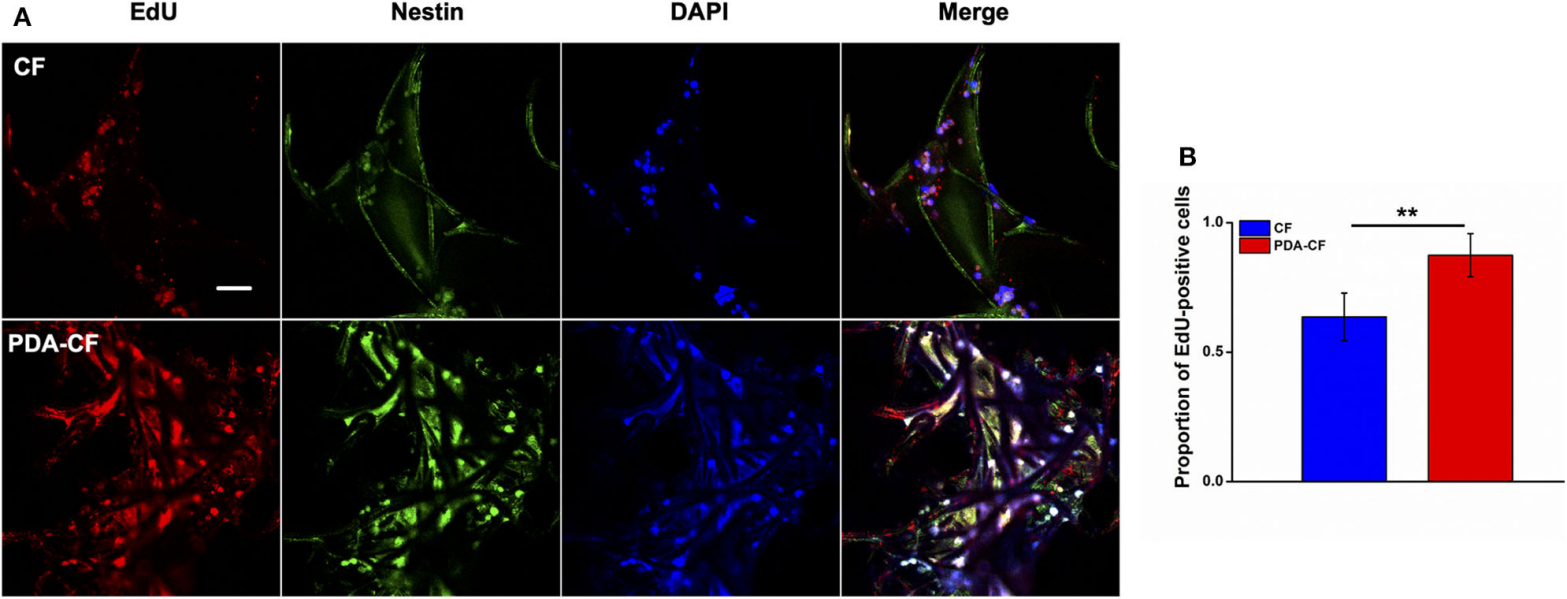
Immunofluorescence staining of EdU and nestin in NSCs cultured for 3 days on the CF and PDA-CF scaffolds. **(A)** The proliferation of NSCs assessed by EdU staining (red), with nestin (green) indicating NSCs. **(B)** Quantification of the EdU-positive NSCs cultured on CF and PDA-CF. Scale bar is 50 μm. The data are presented as the mean ± SD; *p < 0.05, **p < 0.01.

The percentage of EdU-positive cells (Figure 6A) also showed that NSCs grew better on the PDA-functionalized scaffold. After the NSCs were cultured for 1 day, the NSCs were treated with 10 μM EdU to label the mitotic cells. To quantify the proliferating NSCs, we stained the NSCs for the stem cell marker nestin. After 3 days, the number of EdU-positive NSCs on the PDA-CF scaffold was significantly greater than on the CF scaffold. Quantification of the EdU-positive cells (Figure 6B) showed that the mean proportion of EdU-positive cells on the PDA-CF scaffold was 87.43%, but was only 68.9% on the CF scaffold. These results suggested that PDA-CF could substantially promote the proliferation of NSCs.

### Change in Expression of Adhesion Factors

**Figure 7:**
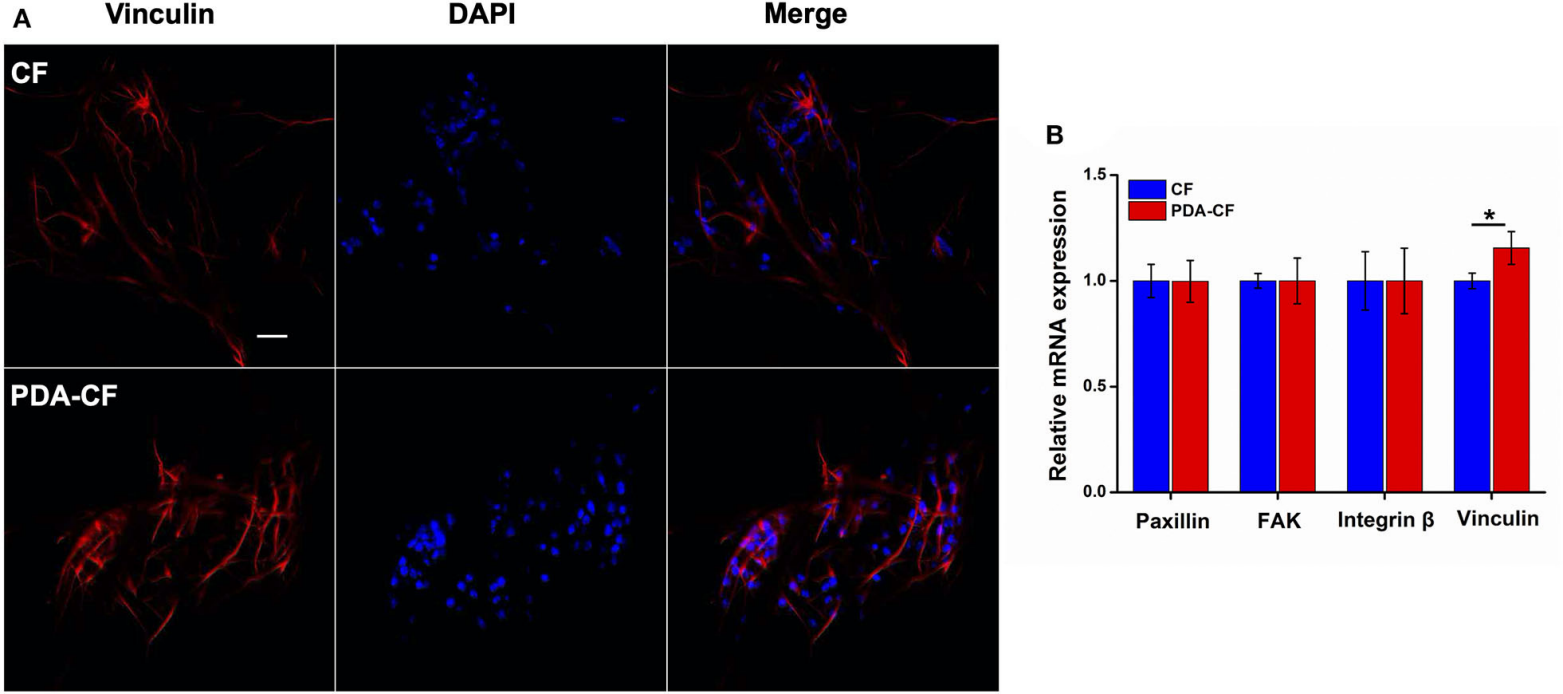
Immunofluorescence images and adhesion molecule gene expression in NSCs cultured on CF and PDA-CF for 3 days. **(A)** NSCs were stained with vinculin (red) and DAPI (blue), and the images were merged. **(B)** mRNA expression of paxillin, FAK, integrin, and vinculin in NSCs cultured on CF and PDA-CF. Only vinculin was significantly up-regulated after culturing on PDA-CF. Scale bar is 50 μm. The data are presented as the mean ± SD; *p < 0.05, *n* = 3.

Cell adhesion plays an essential role in regulating cell proliferation, differentiation, migration, and apoptosis. The SEM micrographs in Figure 3D show that NSCs adhered well to the scaffold surface. The association between NSCs proliferation processes and adhesion factor expression needs to be verified. To assess NSC adhesion, the gene expression of paxillin, focal adhesion kinase (FAK), integrin β, and vinculin was measured by quantitative real-time PCR using GAPDH as the control. Vinculin is a cytoskeletal protein associated with focal adhesion that regulates cell proliferation (Bays and DeMali, 2000). For NSCs cultured on PDA-CF for 3 days, immunohistochemistry showed increased expression of vinculin protein (Figure 7A), and quantification of vinculin (Figure 7B) showed increased expression compared to the CF group, which suggests that PDA might promote NSC proliferation by regulating the expression of vinculin. The interaction between integrins and the ECM induces the expression of paxillin, which is a central component of focal adhesion complexes and plays a vital role in cell attachment, spreading, and migration through the transduction of extracellular signals into intracellular responses (Lo'pez-Colome et al., 2017; Ma and Hammes, 2018). Cells attach to the ECM through trans-membrane integrin proteins and use these interactions to sense their environment (Kechagia et al., 2019). We measured the expression of integrin β by quantitative real-time PCR, and we found no significant changes between the two groups, which means that the proliferation of NSC on PDA-CF has no relationship with this biomolecule synthesis. In addition, we also assayed the expression of FAK, which is a cytoplasmic non-receptor tyrosine kinase that enables activation by growth factor receptors or integrins in various types of human cancers. It is a critical regulator in promoting cancer proliferation (Bullard Dunn et al., 2010; Tai et al., 2015). While from the results, the characteristics of PAD-CF scaffold seems to have no effect on the expression of FAK. In conclusion, PDA-CF might influence vinculin protein and thus regulate NSC proliferation.

In addition, we explored the effect of the PDA-CF scaffold material on the differentiation of NSCs using βIII-tubulin (TuJ1), the expression of which has been widely used as a marker of early neuron development (Ambasudhan et al., 2011). We measured the immunofluorescence staining of TuJ1 at 1 day, 5 days, and 10 days after NSC seeding, and during the period of differentiation detection TuJ1 was expressed weakly in both the PDA-CF and CF groups and there was no significant difference between the two groups (Supplementary Figure 2A). Meanwhile, quantitative analysis of the NSC differentiation-related genes *Gap, Map2*, and *TuJ1* indicated that there was no significant difference in the expression levels between the various genes in cells grown on the two scaffolds (Supplementary Figure 2B). As a result, NSCs did not differentiate into neural cells on the PDA-CF scaffold any more so than on the CF control scaffold.

## Conclusion

The difficulty in achieving functional recovery after nerve injury results in overwhelming medical costs and psychological burden, and thus there is a great need for methods to prevent neuronal cell loss and to promote nerve repair. The replacement of autologous nerves by nerve cells generated through NTE is a current research focus, and the development of biomimetic ECM is also of great importance. In addition, electrical stimulation of neurons in the absence of topographical features has been shown to guide axonal extension. In this paper, we have developed a low-cost and straightforward method for modifying 3D carbon microfibrous scaffolds with PDA to create a microenvironment that is more conducive to cell growth.

The first aim of this research was to fabricate and characterize a PDA-functionalized carbon microfibrous electrospun scaffold. The cellulose was made into a 3D network structure by electrospinning, and it maintained its electrical conductivity and mechanical flexibility and biocompatibility after heat treatment. The second part of this research was to evaluate the material's effects on the proliferation and differentiation of NSCs from the hippocampus of fetal mice. Our results suggest that PDA-CF acts as a neural scaffold that promotes the maturation of NSCs by accelerating cell development and adhesion as indicated by increased Ki-67 expression. Representative 3D reconstruction of confocal microscope images showed that NSCs grew quickly into the interior of the PDA-CF, while only expanding on the surface of the CF scaffold. In addition, the expression of vinculin was significantly enhanced on PDA-CF compared with the CF control group. This is very interesting, in some way, PDA seems to accelerating the development of NSCs by promoting the expression of adhesion protein vinculin.

These results indicated that PDA plays an essential role in promoting the spread of NSCs on the electrically conductive 3D CF skeleton and that the viscous microfibrous networks formed by PDA-CF are key for improving NSC attachment and development. However, the molecular mechanisms behind these effects need to be studied further. NSCs did not differentiate into neural cells on PDA-CF any more so than they did on the CF control material, and we suspect that the reason for this is that NSCs maintained a state of self-renewal in the short time for which they were cultured on PDA-CF. Future work should extend the incubation time of NSCs to 3 weeks in order to further explore the differentiation of cells cultured on PDA-CF scaffolds. This method can be used to prepare multifunctional neuron scaffolds for nerve transplantation treatment and *in vitro* research, and these hybrid materials are also likely to find use with myocardial and other muscle cells to create tissue constructs with improved organization, electroactivity, and mechanical integrity.

## Data Availability Statement

The raw data supporting the conclusions of this article will be made available by the authors, without undue reservation, to any qualified researcher.

## Ethics Statement

The studies involving animal experiments were reviewed and approved by Care and Use of Animals Committee of Southeast University. The study does not involve any human sample or human data.

## Author Contributions

RC, ZG, and YY designed experiments. YY and YZ carried out experiments and analyzed experimental results. YY wrote the manuscript. All authors contributed to the article and approved the submitted version.

## Conflict of Interest

The authors declare that the research was conducted in the absence of any commercial or financial relationships that could be construed as a potential conflict of interest.
