# Supplementary material for: A Polydopamine-Functionalized Carbon Microfibrous Scaffold Accelerates the Development of Neural Stem Cells
Source: Front Bioeng Biotechnol. 2020 Jun 23;8:616. doi: 10.3389/fbioe.2020.00616 (PMC7344254; doi:10.3389/fbioe.2020.00616)
Supplement: Supplementary file 1 [file Table_1.DOCX]

Supplementary Material


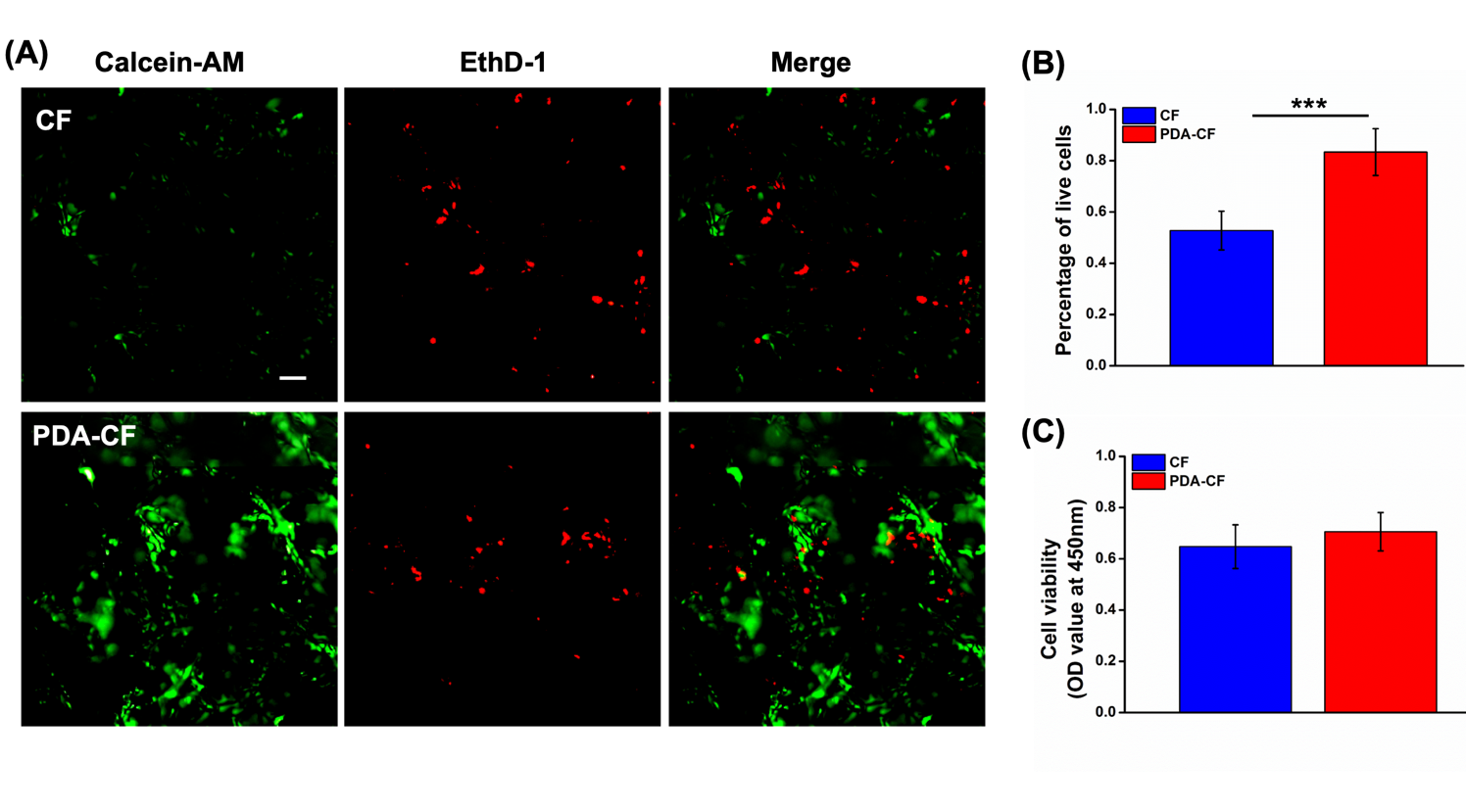


**Supplementary Figure 1.** (A). Viability/cytotoxicity observed in fluorescence images captured 48 hours after seeding. Calcein-AM–stained healthy cells are shown in green, and EthD-1–stained nuclei of dead cells are shown in red. (B) The percentage of live cells on the CF and PDA-CF scaffolds for 48h. Data are presented as the mean ± SD of three replicates. NSCs were seeded at a concentration of 0.6 × 10^6^ cells/ml. (C) The cell viability of NSCs grown on CF and PDA-CF was tested by CCK-8 assay. Scale bar is 80 µm.


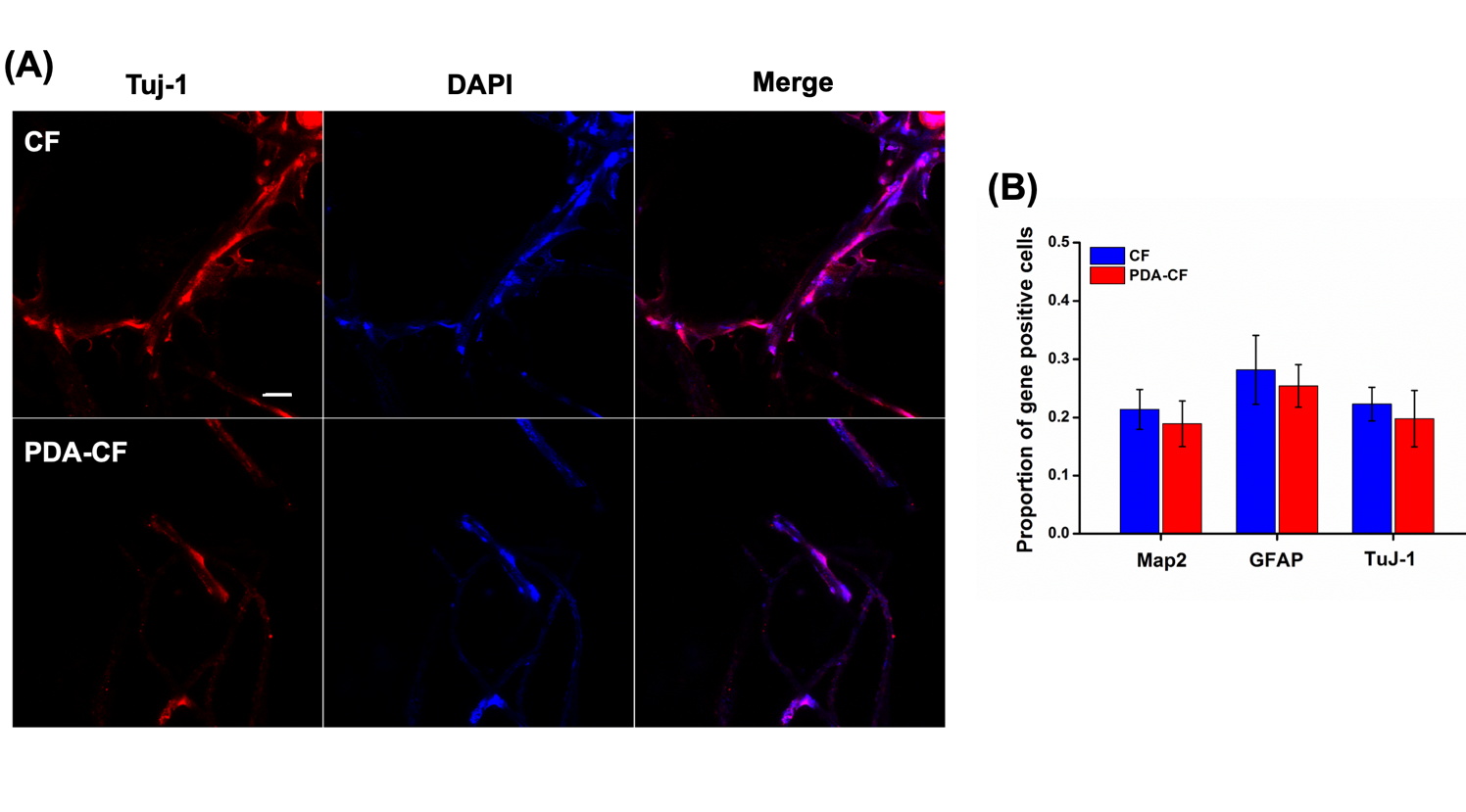


**Supplementary Figure 2.** Immunofluorescence stainings of differentiated neural-associated proteins Tuj-1 (A) and quantification of the Tuj-1-positive NSCs cultured on CF and PDA-CF. (B) after 10 days of culture. Scale bar is 50 µm. The data are presented as the mean ± SD; * p < 0.05, n = 3.
